# Intron losses and gains in the nematodes

**DOI:** 10.1186/s13062-022-00328-8

**Published:** 2022-06-05

**Authors:** Ming-Yue Ma, Ji Xia, Kun-Xian Shu, Deng-Ke Niu

**Affiliations:** 1 Chongqing Key Laboratory of Big Data for Bio Intelligence, School of Bioinformatics, Chongqing University of Posts and Telecommunications, Chongqing, 400065 China; 2 MOE Key Laboratory for Biodiversity Science and Ecological Engineering and Beijing Key Laboratory of Gene Resource and Molecular Development, College of Life Sciences, Beijing Normal University, Beijing, 100875 China

**Keywords:** Intron gain, Intron loss, Nematoda, *Caenorhabditis elegans*, Phylogenetic

## Abstract

**Background:**

The evolution of spliceosomal introns has been widely studied among various eukaryotic groups. Researchers nearly reached the consensuses on the pattern and the mechanisms of intron losses and gains across eukaryotes. However, according to previous studies that analyzed a few genes or genomes, Nematoda seems to be an eccentric group.

**Results:**

Taking advantage of the recent accumulation of sequenced genomes, we extensively analyzed the intron losses and gains using 104 nematode genomes across all the five Clades of the phylum. Nematodes have a wide range of intron density, from less than one to more than nine per kbp coding sequence. The rates of intron losses and gains exhibit significant heterogeneity both across different nematode lineages and across different evolutionary stages of the same lineage. The frequency of intron losses far exceeds that of intron gains. Five pieces of evidence supporting the model of cDNA-mediated intron loss have been observed in ten *Caenorhabditis* species, the dominance of the precise intron losses, frequent loss of adjacent introns, high-level expression of the intron-lost genes, preferential losses of short introns, and the preferential losses of introns close to 3′-ends of genes. Like studies in most eukaryotic groups, we cannot find the source sequences for the limited number of intron gains detected in the *Caenorhabditis* genomes.

**Conclusions:**

These results indicate that nematodes are a typical eukaryotic group rather than an outlier in intron evolution.

**Supplementary Information:**

The online version contains supplementary material available at 10.1186/s13062-022-00328-8.

## Background

In the nuclear genomes, protein-coding genes are often interrupted by noncoding sequences removed from the pre-mRNAs by the dynamic RNA–protein complex, spliceosome. In most publications, these interrupting sequences are termed spliceosomal introns and abbreviated as introns. Eukaryotic genomes vary considerably in their intron contents. The human genome contains hundreds of thousands of introns, with each human gene having eight introns on average [1]. The dinoflagellate *Symbiodinium minutum* has an even higher intron density in its genome, with up to 18.6 introns per gene [2]. On the other side, the yeast *Saccharomyces cerevisiae* genes have only 0.05 introns on average. Furthermore, the highly compacted genomes of some obligate intracellular microbes do not have any introns [3, 4]. A large-scale comparative analysis showed that the ancestors of all major eukaryotic groups and the last eukaryotic common ancestor all have intron-rich genomes, with the intron densities ranging from 53 to 74% of that in the human genome [5]. Together with this one, many studies indicate that recurrent intron losses dominated the evolution of eukaryotic genes, with a few episodes of substantial gains [6–21].

The differential rates of intron loss and gain across eukaryotic lineages result from the differences in the rates of spontaneous mutations giving rise to new intron loss or gain events and the probability of fixing the new mutations in the genomes. Introns allow one gene to code multiple proteins through alternative splicing [22, 23]. Some intron sequences are recruited as regulatory elements of gene expression or harbor functional noncoding RNAs, and the splicing process might benefit the organisms by preventing DNA damage associated with transcription [24–30]. Although there has been much agreement that a fraction of introns has essential biological functions, we wonder whether the rest positively affects the organisms. The most solid evidence on the beneficial impact of introns comes from the intron-poor eukaryote, *S. cerevisiae* [31–34]. It is very likely that the yeast genome has experienced extensive intron losses and retained only the introns that have functional roles or acquired some beneficial effects by processes like constructive neutral evolution [35].

On the other hand, most introns have been suggested to be slightly deleterious [36–38]. Thus, the fixation of the intron loss/gain events depends on natural selection efficiency, mainly determined by the effective population size. However, this hypothesis was not supported by analyzing the intron gains in the genomic regions with reduced election efficiency across major eukaryotic lineages [39]. Instead, Roy [39] advocated an alternate explanation. The availability of spontaneous mutations giving rise to new introns or removing old introns might drive the evolution of intron–exon structures, while selective differences play only a minor role. Consistent with this idea, massive intron gains were observed only in the genomes containing a family of transposable elements that carry splicing signals [16–19, 40–43]. Meanwhile, intron loss frequency is associated with reverse transcriptase activity [44–47].

The most widely cited mechanism of intron loss is recombining the genomic DNA with the cDNA molecules reverse-transcribed from mature mRNAs [1, 48]. Evidence supporting this idea, including precise intron loss, simultaneous loss of adjacent introns, preferential loss of short intron, and biased loss of introns at the 3′ side of genes have been repeatedly reported in most studied on eukaryotic genome evolution, from protists, fungi, plants to animals [6, 20, 49–56].

However, previous studies showed an entirely different picture of intron evolution in the nematodes. Phylogenetic analyses of a few genes or gene families found that the vast majority of intron changes during nematode evolution involve losses of introns individually, rather than multiple introns being lost together [57, 58]. The authors advocated an alternate hypothesis. An intron could be simply lost in a mutation of genomic deletion, possibly involving nonhomologous recombination stimulated by the existence of short direct repeats at or near the two ends of an intron. Besides the individual loss of introns, this hypothesis predicts that most intron losses are not precise deletion of introns from genomic DNA but accompanied by the insertion and/or deletion (indel) of a few nucleotides into/from the flanking exons. The eccentricity of nematode intron losses was further strengthened by analyzing the genome-wide alignments of *Caenorhabditis elegans* and *C. briggsae* [59]. It is impossible to distinguish intron losses from intron gains from the alignments between orthologous sequences of just two species. However, referring to the previous results, the authors believed that most of the intron changes they observed were intron losses. In total, they observed 263 changes of exact intron changes. Meanwhile, they detected 518 intron changes that caused indels to the flanking exons. Their results suggested that imprecise intron losses outnumbered precise intron losses in nematodes. Later, Roy and Gilbert studied the intron losses in 684 groups of orthologous genes from seven eukaryotes, including *Homo sapiens*, *Drosophila melanogaster*, *Anopheles gambiae*, *C. elegans*, *Schizosaccharomyces pombe*, *Arabidopsis thaliana*, and *Plasmodium falciparum*. They observed evidence supporting the cDNA-mediated intron loss model, biased loss from 3′-end and adjacent intron loss. However, none of these patterns were observed in *C. elegans*, leading them to conclude that the intron loss process might be qualitatively different in nematodes [50]. The lacking of evidence supporting the model of cDNA-mediated intron loss in the nematodes was further strengthened by another study of five *Caenorhabditis* genomes [60].

On the other side, the studies on intron gain took an unexpected turn in the nematodes. Coghlan and Wolfe [61] compared the intron–exon structures between *C. elegans* and *C. briggsae* using the distantly related nematode *Brugia malayi*, two chordates (human and mouse), and two arthropods (fruit fly and mosquito) as outgroups. They found 122 recently gained introns in the two nematode genomes, and 28 of them have significant sequence identity to other introns, providing evidence for the introns’ origin. Roy and Penny [13] repeated the study 2 years later using two newly sequenced relatives: *C. remanei* and *Caenorhabditis* sp. 4. Their results showed that most of the 122 intron gains reported in one *Caenorhabditis* species are actually intron losses in other species [13]. This result highlights the importance of the dense phylogenetic sampling of closely related species to draw accurate inferences about intron evolution [62].

All the previous studies on nematode intron evolution were based on a few gene families or genomes whose sequences were available at that time. With the rapid progress of genome sequencing and annotation, nearly 200 completely sequenced genomes are now available in WormBase [63]. It is time to comprehensively revisit the nematode intron evolution based on a dense phylogenetic sampling of closely related genomes. Using 104 nematode genomes, we carried out an extensive study on nematode intron evolution, with the molecular mechanism of intron loss in the *Caenorhabditis* branch deeply investigated.

## Results

### The phylogenetic tree of the nematode species

**Fig. 1:**
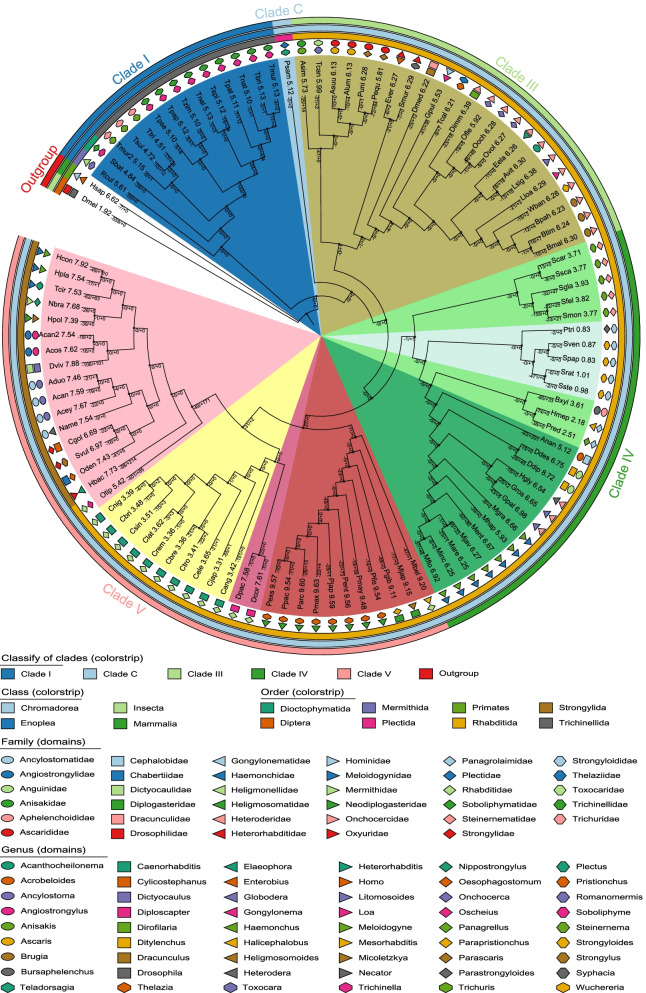
Intron losses and gains during the evolution of nematodes. The present tree is the best one obtained in the maximum likelihood analysis of 1551 groups of orthologous protein alignments. The number of intron losses and gains of each branch was computed by the maximum likelihood with the rate-variation model of MALIN [65]. The values are displayed on the branch lines, using “+” and “−” symbols to represent intron gain and intron loss, respectively. The numbers behind species names are intron densities. Please see Additional file 1: Table S2 for the full name of each species and the values present in this figure. Sister figures (Additional file 2: Fig. S1 and S2) showing the ancestral intron densities and the rates of intron losses and gains are deposited in Additional file 2

Using the best reciprocal basic local alignment search tool for protein (BLASTP) hits with a threshold E value of 10^−5^, we captured the 1557 groups of orthologs that are present in over 90% of the analyzed species (104 nematode species and two outgroup species, *D. melanogaster* and *H. sapiens*), and at least in one of the two outgroup species. After filtration of the poorly aligned regions from the multiple sequence alignments, 1551 groups of orthologous genes were obtained. A molecular consensus tree was constructed using these orthologous genes (Fig. 1). Only one node bootstrap value was 86. The others were more than 90, even most of the values (93.2%) were equal to 100. Each of the five major clades identified by Blaxter et al. [64] and adopted by the database WormBase [63] were distinctively clustered in the phylogenetic tree we constructed (Fig. 1).

### Intron densities of modern nematodes and ancestral nematodes

We first calculated the intron density, the intron number per 1 kbp coding sequence (CDS), of the 1577 groups of orthologs across the 106 genomes. The intron density values of the model organisms we obtained are consistent with the previous study, with *C. elegans*, *D. melanogaster*, and *H. sapiens* having 3.65, 1.92, and 6.62, respectively [5]. The intron densities of the modern nematode genomes have a wide range, from less than one to more than nine (Fig. 1 and Additional file 1: Table S1). Considerable differences in intron density were observed among Clade IV and Clade V species but not Clade I or Clade III (Fig. 1). The most intron-poor family Strongyloididae with intron densities ranging from 0.83 to 1.01, appears in Clade IV. At the same time, other lineages of the same clade have intron densities 2.18 to 8.72, with a median value of 6.25. The most intron-rich group, with a median value of intron density up to 9.54, is the basal taxa of the Clade V, including eight species of the family Neodiplogasteridae and three other species, *Micoletzkya japonica*, *Parapristionchus giblindavisi*, and *Mesorhabditis belari*. The well-studied genus *Caenorhabditis* is also presented in the Clade V. The ten *Caenorhabditis* species have a striking difference in intron density with other species in Clade V, with the median values 3.42 *vs*. 7.68.

We then reconstructed ancestral intron densities (Additional file 2: Fig. S1) and obtained the value of 10.22 introns/kbp for the last common nematode ancestor. It was slightly higher than animal ancestor (8.8 introns/kbp) [21] and close to modern intron-rich nematode species. The model species in *Caenorhabditis* (from 3.31 to 3.65) have evolved to nearly 1/3 of the ancestral intron density, while intron-poor species in Strongyloididae have less than 1/10 of ancestral intron density.

### Intron evolution dynamics during nematode evolution

We estimated ancestral intron content and calculated the number of intron losses and gains on each phylogenetic branch using MALIN [65]. The first pattern of nematode intron evolution that we can see is that intron losses are more frequent than intron gains (Fig. 1 and Additional file 1: Table S2). The total number of intron losses during nematode evolution (4070) was nearly about two times of intron gains (2291). Among the 207 branches, there were only 27 branches where the intron gains outnumbered intron losses. Wilcoxon signed rank test (2-tailed) showed that the difference was highly significant (*p* = 8.0 × 10^−10^). The second pattern we observed in nematode intron evolution is that the lineages with a higher intron loss rate generally have a higher gain rate (Fig. 1, Additional file 1: Table S2, and Additional file 2: Fig. S2). Although this pattern is not so evident as the first one, statistical analysis showed that the positive correlation between the rate of intron loss and intron gain across the 207 branches is highly significant (Spearman′s rho = 0.55, *p* = 9.7 × 10^−18^). The third pattern we could see from Fig. 1 is the vast heterogeneity in intron gain and loss rates across lineages and historical stages of the same lineage (Fig. 1). For example, the two families, Meloidogynidae and Strongyloididae, presented within the same group, Clade IV, experienced entirely different dynamics of intron evolution. High frequencies of intron loss and gain constantly occurred with the lineage splitting during the evolution of the family Meloidogynidae, whereas only four intron losses and no intron gains happened in the family Strongyloididae.

Among the 207 branches during nematode evolution, the intron density are negatively correlated with the number (Spearman′s rho =  − 0.153, *p* = 0.028) and rate (Spearman′s rho =  − 0.240, *p* = 0.001) of intron loss, and the intron gain number (Spearman′s rho =  − 0.218, *p* = 0.002), but not correlated with intron gain rate (Spearman′s rho = 0.119, *p* = 0.088). In addition, we found that the branch length is positively correlated to intron loss (Spearman′s *rho* = 0.282, *p* = 4.0 × 10^−5^), but not correlated with with intron density or intron gains (Spearman′s rho =  − 0.043, *p* = 0.542).

### Intron variations among the 104 nematodes

To evaluate the phylogenetic effect in nematode evolution analysis, we first calculated the phylogenetic signals: *λ* = 0.99 (*p* = 2.4 × 10^−14^), 0.95 (*p* = 1.9 × 10^−5^), and 1.00 (*p* = 3.6 × 10^−59^) for the intron losses, the intron gains, and present intron density, respectively. It seems that phylogenetic comparative methods are required to control the effects of common ancestors. We used the phylogenetic generalized least squares (PGLS) regression analysis to examine the relationships. A positive slope of the regression line indicates a positive correlation, while a negative slope indicates a negative correlation. Consistent with that observed in analyzing the branches, the number of intron losses of the 104 nematodes is positively correlated with that of intron gains (slope = 0.637, *p* = 5 × 10^−15^; Table 1).

**Table 1:** Relationships among the frequencies of intron losses and intron gains, and genomic characteristics in 104 nematodes species

*y*	*x*	Slope	*P*	*P*_BH_	*R*^*2*^
Intron losses	Intron gains	0.712	5 × 10^−15^	6 × 10^−14^	0.4485
	Intron density	− 7.207	0.001	0.004	0.0914
	Genome size	− 0.029	0.540	0.669	− 0.0061
	CDS length	− 0.036	0.024	0.061	0.0399
	Exon length	− 0.004	0.986	0.986	− 0.0098
	Intron length	− 0.043	0.479	0.650	− 0.0048
	Coding gene number	0.000	0.817	0.885	− 0.0093
	Total intron number	− 0.002	0.041	0.089	0.0308
Intron gains	Intron density	− 4.761	0.012	0.039	0.0509
	Genome size	0.004	0.921	0.958	− 0.0097
	CDS length	− 0.035	0.025	0.061	0.0388
	Exon length	− 0.289	0.143	0.286	0.0113
	Intron length	0.026	0.594	0.702	− 0.0070
	Coding gene number	0.000	0.500	0.650	− 0.0053
	Total intron number	− 0.001	0.218	0.354	0.0052
Intron density	Genome size	0.005	0.026	0.061	0.0383
	CDS length	0.004	6 × 10^−11^	5 × 10^−10^	0.3376
	Exon length	− 0.053	10^−7^	7 × 10^−7^	0.2359
	Intron length	0.002	0.491	0.650	− 0.0051
	Coding gene number	10^−5^	0.167	0.310	0.0091
	Total intron number	4 × 10^−4^	< 2 × 10^−16^	5 × 10^−15^	0.6234
Genome size	CDS length	0.028	0.376	0.575	− 0.0020
	Exon length	− 0.230	0.662	0.748	− 0.0079
	Intron length	0.483	2 × 10^−4^	0.001	0.1158
	Coding gene number	0.002	9 × 10^−7^	5 × 10^−6^	0.2037
	Total intron number	0.002	0.209	0.354	0.0058

The relationships were analyzed using phylogenetic generalized least squares analysis. The phylogenetic signals (*λ*) are 0.99 (*p* = 2.4 × 10^−14^), 0.95 (*p* = 1.9 × 10^−5^), 1.00 (*p* = 5.6 × 10^−46^), 1.00 (*p* = 3.4 × 10^−23^), 0.82 (*p* = 5.0 × 10^−14^), 1.00 (*p* = 2.1 × 10^−54^), 1.00 (*p* = 2.7 × 10^−47^), 0.54 (*p* = 7.6 × 10^−8^), and 0.97 (*p* = 1.4 × 10^−32^) for intron losses, intron gains, scaled intron density (intron density), genome size, the median length of protein coding sequences (CDS length), the median length of exon (exon length), the median length of intron (intron length), coding gene number, and total intron number, respectively. Except for the genome size, these traits were calculated from the 1577 orthologs of each genome. *P* and *R*^2^, the *p*-value and adjusted R-squared obtained in phylogenetic generalized least squares analysis.; *P*_BH_, the *p*-value adjusted by Benjamini–Hochberg

Furthermore, we examined the relationship of intron losses/gains with current intron density and other genomic features (Table 1). Intron loss and gain are negatively correlated with intron density but not with other genomic features like genome size and CDS length. The intron density is positively related to genome size, coding sequence length, total intron number but negatively related with exon length (Table 1).

Multiple correlation analyses have been performed based on the same dataset so that some results might be significant by chance. Therefore, we controlled the false discovery rate using the Benjamini–Hochberg (BH) procedure and provided the adjusted *p* values in Table 1. The conclusions presented above are not changed after these corrections except the correlations of the CDS length with the intron losses and intron gains and the correlation between intron density and genome size.

### Intron variations in *Caenorhabditis*

To gain insight into the mechanism of intron losses and gains in nematodes, we analyzed the ten *Caenorhabditis* species in-depth. Using BLASTP (threshold of *E* value = 10^−10^), we identified 4892 groups of orthologous genes present in all ten species. Among them, 6441 discordant intron positions were detected in 2333 groups of orthologs. Meanwhile, 6252 conserved intron positions were identified. Some ambiguous intron positions were discarded. In 682 groups of orthologs, all the intron positions are conserved. Referring to the phylogenetic tree of the ten *Caenorhabditis* species and 12 outgroup species (Additional file 2: Fig. S3), we identified 5047 cases of intron loss and 262 cases of putative intron gain in the ten *Caenorhabditis* species. To avoid the mis-annotations of new insertions in the transcripts into novel introns, we corrected the exon–intron structures of the putative intron-gained genes using RNA-Seq data. In this way, the annotations of 168 novel introns were confirmed. Although the sample size is too small to give statistical conclusions, two evident patterns could be seen. The first is that intron loss frequency is superior to intron gain (Table 2). The second is a positive association between the number of intron losses and that of intron gains. The highest number of intron losses and the highest number of intron gains were detected in the basal lineage, *C. angaria* (Table 2). These results are consistent with the above analysis across the nematodes.

**Table 2:** Intron losses and gains in *Caenorhabditis*

Species	Intron losses	Intron-lost genes	Intron gains	Intron-gained genes	Gene with both intron loss and gain
*C. angaria*	2981	1534	130	118	80
*C. japonica*	918	672	16	15	7
*C. elegans*	329	274	13	13	3
*C. tropicalis*	279	246	*	−	−
*C. brenneri*	256	225	7	6	4
*C. latens*	41	38	0	−	−
*C. remanei*	31	27	0	−	−
*C. sinica*	166	142	*	−	−
*C. nigoni*	39	35	0	−	−
*C. briggsae*	7	6	2	2	0
Sum	5047	3199	168	154	94

*We detected six putative intron gains in *C. tropicalis* and 12 in *C. sinica*. However, none of their annotations were confirmed by the RNA-seq data and thus, they were not counted as novel introns

Moreover, we noticed some genes that experienced both intron losses and intron gains (Table 2). Among the 4892 orthologous genes in *C. angaria*, the intron-lost genes (1534) take 31.4%, and the intron-gained genes (118) take 2.41%. If intron losses and gains are randomly distributed among the genes, the genes that experienced both intron losses and intron gains should take 0.76%, i.e., 37 genes. This expected number is significantly smaller than the observed 80 genes (Pearson′s *Chi-squared* test, *p* = 6 × 10^−18^). The same patterns were observed in *C. japonica*, *C. elegans*, and *C. brenneri* (Pearson′s *Chi-squared* test, *p* = 2 × 10^−4^, 0.006, and 4 × 10^−13^, respectively).

In all the ten species, the CDSs of the intron-lost genes are consistently longer than those of the intron-conserved genes (Table 3). Although the BH correction for multiple comparisons is often suggested to be too conservative and might lead to false-negative results, all the BH-adjusted *p* values are smaller than 0.05. We also found that the intron-lost genes are consistently longer than the intron-conserved genes in all the ten species (Mann–Whitney *U* test, BH-adjusted *p* < 0.05 for all cases, Table 3). It should be noted that the lengths of the lost introns were not counted in calculating the size of intron-lost genes. Therefore, this is a stringent comparison to test the hypothesis that long genes are more likely to lose their introns than short genes.

**Table 3:** Comparing the gene structures between the intron-lost genes and intron-conserved genes in *Caenorhabditis*

Species	Gene type	Gene number	Coding sequence length		Gene length		Intron number	
			Median (bp)	*p*-value	Median (bp)	*p*-value	Median	*p*-value
Cang	Conserved	682	767	*P*_U_ = 10^−67^	1791	*P*_U_ = 7 × 10^−30^	3	*P*_U_ = 2 × 10^−30^
	Lost	1534	1350	*P*_BH_ = 10^−66^	2785	*P*_BH_ = 3 × 10^−29^	5	*P*_BH_ = 2 × 10^−29^
Cjap	Conserved	682	899	*P*_U_ = 2 × 10^−56^	2860	*P*_U_ = 6 × 10^−31^	4	*P*_U_ = 4 × 10^−24^
	Lost	672	1572	*P*_BH_ = 9 × 10^−56^	4842	*P*_BH_ = 6 × 10^−30^	6	*P*_BH_ = 2 × 10^−23^
Cele	Conserved	682	1022	*P*_U_ = 4 × 10^−32^	2673	*P*_U_ = 5 × 10^−15^	5	*P*_U_ = 3 × 10^−13^
	Lost	274	1754	*P*_BH_ = 10^−31^	4375	*P*_BH_ = 10^−14^	7	*P*_BH_ = 8 × 10^−13^
Ctro	Conserved	682	945	*P*_U_ = 2 × 10^−28^	1586	*P*_U_ = 10^−14^	4	*P*_U_ = 3 × 10^−12^
	Lost	246	1623	*P*_BH_ = 3 × 10^−28^	2480	*P*_BH_ = 3 × 10^−14^	6	*P*_BH_ = 6 × 10^−12^
Cbre	Conserved	682	987	*P*_U_ = 3 × 10^−33^	1956	*P*_U_ = 2 × 10^−15^	5	*P*_U_ = 4 × 10^−14^
	Lost	225	1797	*P*_BH_ = 9 × 10^−33^	3031	*P*_BH_ = 8 × 10^−15^	6	*P*_BH_ = 10^−13^
Clat	Conserved	682	999	*P*_U_ = 10^−7^	2098	*P*_U_ = 9 × 10^−6^	5	*P*_U_ = 10^−5^
	Lost	38	1733	*P*_BH_ = 2 × 10^−7^	3742	*P*_BH_ = 10^−5^	8	*P*_BH_ = 2 × 10^−5^
Crem	Conserved	682	1010	*P*_U_ = 5 × 10^−5^	2255	*P*_U_ = 0.0005	5	*P*_U_ = 0.0007
	Lost	27	1488	*P*_BH_ = 6 × 10^−5^	3695	*P*_BH_ = 0.0006	7	*P*_BH_ = 0.0008
Csin	Conserved	682	987	*P*_U_ = 5 × 10^−22^	1893	*P*_U_ = 2 × 10^−12^	5	*P*_U_ = 3 × 10^−12^
	Lost	142	1794	*P*_BH_ = 8 × 10^−22^	3111	*P*_BH_ = 4 × 10^−12^	7	*P*_BH_ = 6 × 10^−12^
Cnig	Conserved	682	1017	*P*_U_ = 10^−5^	2260	*P*_U_ = 0.0078	5	*P*_U_ = 0.0002
	Lost	35	1584	*P*_BH_ = 10^−5^	2889	*P*_BH_ = 0.0080	8	*P*_BH_ = 0.0002
Cbri	Conserved	682	1040	*P*_U_ = 0.0045	2714	*P*_U_ = 0.0080	5	*P*_U_ = 0.0141
	Lost	6	1880	*P*_BH_ = 0.0045	5108	*P*_BH_ = 0.0080	8	*P*_BH_ = 0.0141

*Cang* *C. angaria*, *Cjap* *C. japonica*, *Cele* *C. elegans*, *Ctro* *C. tropicalis*, *Cbre* *C. brenneri*, *Clat* *C. latens*, *Crem* *C. remanei*, *Csin* *C. sinica*, *Cnig* *C. nigoni*, *Cbri* *C. briggsae*, conserved intron-conserved genes, lost intron-lost genes, *P*_*U*_ the *p*-value obtained in Mann–Whitney *U* test, *P*_*BH*_ the *p-*value adjusted by Benjamini–Hochberg procedure

We propose that long genes might have more introns and thus be more likely to lose some of their introns just by chance. Therefore, we compared the number of introns between intron-lost and intron-conserved genes. As shown in Table 3, the intron-lost genes consistently have more introns than the intron-conserved genes in all the ten *Caenorhabditis* species (Mann–Whitney *U* test, BH-adjusted *p* < 0.05 for all cases). It should be noted that the number of lost introns was not counted in the intron number of the intron-lost genes.

### The mechanism of intron losses in *Caenorhabditis*

Among the 5047 cases of intron loss identified in the *Caenorhabditis* clade, 4844 cases (96%) are precise intron losses. The percentage of accurate intron losses in each genome ranges from 90.2 to 100% (Table 4). In total, there are 828 pairs of adjacent intron losses. Resampling analysis showed that adjacent intron loss frequency is significantly higher than expected by chance in four *Caenorhabditis* species (*p* < 0.05 for all cases, *C. angaria*, *C. japonica*, *C. elegans*, and *C. nigoni*, Table 4).

**Table 4:** Frequency of precise intron losses and adjacent intron losses in *Caenorhabditis*

Species	Intron losses	Precise losses	Genes with ≥ 2 lost introns	Adjacent pairs^a^	*p*-value^b^	*p*-value^c^
*C. angaria*	2981	2866	757	643	−	0
*C. japonica*	918	882	166	119	−	0
*C. elegans*	329	314	43	26	−	10^−4^
*C. tropicalis*	279	269	25	14	0.098	0.085
*C. brenneri*	256	245	28	11	0.153	0.081
*C. latens*	41	37	3	2	0.218	0.110
*C. remanei*	31	30	2	2	0.387	0.361
*C. sinica*	166	158	17	7	0.175	0.124
*C. nigoni*	39	36	3	3	0.116	0.022
*C. briggsae*	7	7	1	1	0.250	0.097

^a^The number of adjacent pairs of intron losses

^b^The probabilities of adjacent intron losses were calculated referring to [50]

^c^The random resampling method was used to calculate the probabilities of adjacent intron losses

**Fig. 2:**
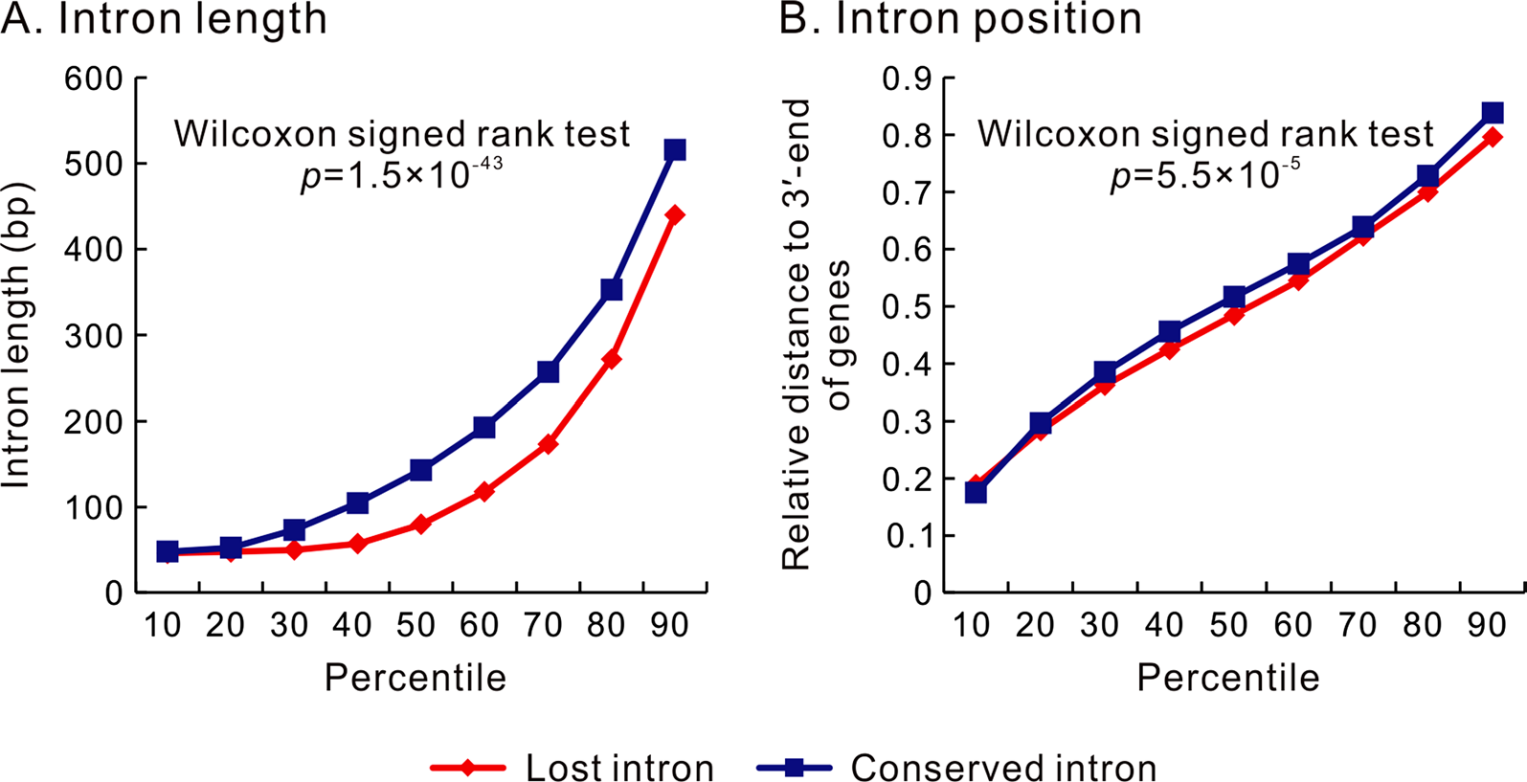
Comparison between the lost introns and conserved introns in *Caenorhabditis*. The 10th to 90th percentiles of the data are presented. **A** The lost introns are significantly shorter than the conserved introns of the same genes. **B** The lost introns are closer to the 3′-ends of genes than conserved introns of the same genes. Three thousand one hundred ninety-nine intron-lost genes in *Caenorhabditis* were used in these two comparisons. The original data for this figure were deposited in and Additional file 1: Table S4

To study the lengths of a lost intron, we had to use the length of its extant ortholog in a closely related species to represent its length. Meanwhile, the lengths of the conserved introns were also represented by the orthologous introns of the same closely related species. The prerequisite for these representations is a significant correlation in intron length between the two closely related species. Our nonparametric rank correlation analyses revealed significant positive correlations for all pairs of *Caenorhabditis* species (*p* < 0.001 for all the cases, Additional file 1: Table S3). Therefore, the length of lost introns could be represented by the size of their orthologous introns in closely related species. However, for early diverged species, like *C. angaria*, the length of lost introns could only be poorly represented by their orthologous introns in other *Caenorhabditis* species. Of course, the latter is also statistically acceptable. In this way, we compared the lengths of lost introns and conserved introns (Table 5). In most *Caenorhabditis* species (7/10), lost introns were significantly shorter than conserved introns (Mann–Whitney *U* test, *p* < 0.05 for all cases). However, in the other three species (*C. latens*, *C. nigoni*, and *C. briggsae*), no statistically significant differences were found (Mann–Whitney *U* test, *p* > 0.05 for all cases). Then, we performed this comparison within the intron-lost genes by combining all the intron-lost genes of the ten species into one large sample. Here, we found that the lost introns are significantly shorter than the extant introns of the same genes (Wilcoxon signed rank test, *n* = 3199, *p* = 1.5 × 10^−43^, Fig. 2A and Additional file 1: Table S4).

**Table 5:** Comparison of the lengths between lost introns and conserved introns

Species	Lost introns (bp)		Conserved introns (bp)		*p*-value	*BH-adjusted p*
	Mean ± SD	Median	Mean ± SD	Median		
*C. angaria*	192 ± 315	86	252 ± 330	139	3 × 10^−44^	3 × 10^−43^
*C. japonica*	184 ± 274	64	226 ± 323	104	7 × 10^−15^	4 × 10^−14^
*C. elegans*	200 ± 316	51	214 ± 318	83	10^−6^	3 × 10^−6^
*C. tropicalis*	151 ± 254	50	225 ± 548	54	7 × 10^−5^	0.0001
*C. brenneri*	88 ± 141	46	154 ± 309	48	10^−11^	3 × 10^−11^
*C. latens*	266 ± 434	49	195 ± 356	53	0.546	0.607
*C. remanei*	107 ± 164	49	186 ± 346	53	0.013	0.019
*C. sinica*	248 ± 701	49	271 ± 540	54	0.002	0.003
*C. nigoni*	677 ± 1547	51	294 ± 842	53	0.914	0.914
*C. briggsae*	239 ± 199	211	247 ± 413	53	0.362	0.453

The numbers of the lost introns in each species are in the second columns of Tables 2 and 4, and the number of the conserved introns was 6252. The length of its ortholog represented the length of a lost intron, and that of orthologous introns represented that of the conserved introns for comparison. The *P*-value was computed by the Mann–Whitney *U* test. BH: Benjamini–Hochberg

To test whether the introns close to the 3′-ends of genes were preferentially lost, we compared the lost introns and the conserved introns for their relative distance to the 3′-ends of genes defined as the ratio of their distances to the 3′-ends of the CDSs divided by the CDS lengths. First, we compared the lost introns with all the conserved introns in the 4892 groups of orthologous genes. Only in two of the ten species (*C. japonica* and *C. remanei*), we find that the lost introns are significantly close to the 3′-ends of genes than the conserved introns (Mann–Whitney *U* tests, BH-adjusted *p* < 0.05 for both cases). Then, we confined this comparison within the intron-lost genes. We averaged the relative distances of the conserved introns and those of the lost introns for each intron-lost gene. Although the lost introns' mean and median values are consistently smaller than those of the conserved introns, statistically significant differences were not observed in any species after the BH correction for multiple comparisons (Wilcoxon signed rank test *p* > 0.05 for all these three cases). However, when all the intron-lost genes from different species are considered together, they exhibit a significant difference: the lost introns are closer to the 3′-ends of genes than the current introns of the same genes, with the median values of the relative distance to the 3′-ends of genes being 0.485 and 0.517, respectively (Wilcoxon signed rank test, *p* = 5.5 × 10^−5^, Fig. 2B and Additional file 1: Table S4).

**Fig. 3:**
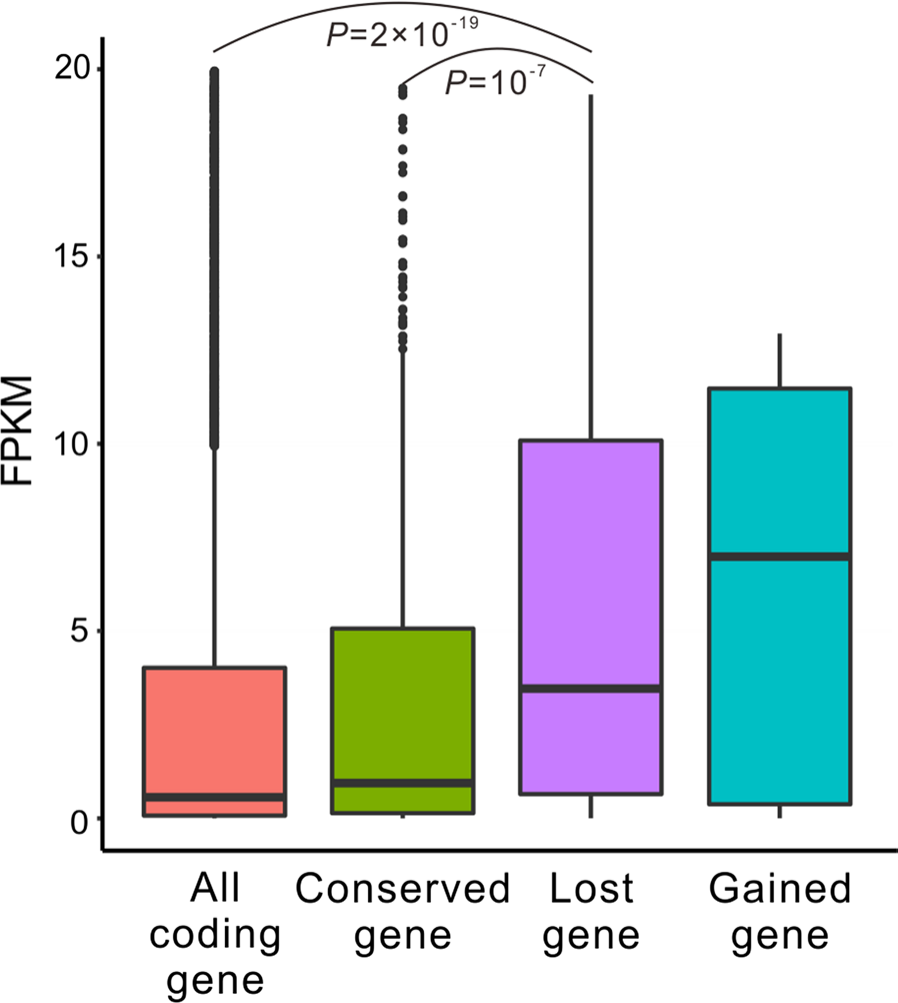
Higher expression level in the intron-lost genes of *C. elegans*. The numbers of all annotated coding genes, intron-conserved genes, intron-lost genes, and intron-gained genes were 16,031, 677, 273, and 13, respectively. The FPKM value represents the gene expression levels, and the FPKM values equal to zero were removed. The FPKM values higher than 25 are not shown in the box charts. The *p*-values of Mann–Whitney *U* tests were 10^−7^ and 2 × 10^−19^, respectively. The small sample size makes comparing intron-gained genes with other genes statistically meaningless. The original data for this figure were deposited in and Additional file 1: Table S5

Finally, we compared the expression levels of the intron-lost genes and the intron-conserved genes using the fragments per kilobase of transcript per million mapped reads (FPKM) values to estimate the gene expression level. The median FPKM values of the intron-lost and intron-conserved genes are 5.81 and 1.62, respectively (Fig. 3 and Additional file 1: Table S5). Mann–Whitney *U* tests showed that the intron-lost genes have significantly higher expression levels than intron-conserved genes (*p* = 10^−7^). As an mRNA with more copies in the cytoplasm is more likely to be reverse-transcribed into cDNA, the higher expression levels of the intron-lost genes could be regarded as another piece of evidence supporting the model of cDNA-mediated intron loss.

### Cases of intron gains in *Caenorhabditis*

From the 6441 discordant intron positions, we identified 168 novel introns (Additional file 1: Table S6). The newly gained introns are not evenly distributed among the ten *Caenorhabditis* species but roughly correlated with the divergence time of each species. The basal branch (*C. angaria*) has 130 new introns. Followed by later diverged species, there are 16 new introns in *C. japonica*, 13 new introns in *C. elegans*, seven new introns in *C. brenneri*, and only two new introns in *C. briggsae*. No new introns were detected in other *Caenorhabditis* species. The 130 novel introns of *C. angaria* were distributed in 118 protein-coding genes, with five pairs of adjacent novel introns. No adjacent novel introns were found in other *Caenorhabditis* species. Hoping to find the possible source sequences for the novel introns, we searched the highly similar sequences of the novel introns in the nr/nt database and the National Center for Biotechnology Information (NCBI) genome database. Unfortunately, no possible source sequences of the new introns were identified.

The features of the 168 newly gained introns were characterized. First, most (166) were gained without causing insertions to or deletions from the flanking exonic sequences. Second, all the 168 new introns have the gt-ag conservative splicing signals and the polypyrimidine tracts. Third, we compared the intron-loss genes with the intron-conserved genes of the same genome. Because *C. angaria* has a statistically large number of intron gains, we compared its intron-gained genes with its intron-conserved genes (118 *vs*. 682) using Mann–Whitney *U* tests. The intron-gained genes have significantly more introns than with more introns than conserved genes (median values: 6 vs. 3, *p* = 4.7 × 10^−18^). The difference was still highly significant even the newly acquired introns were not counted in the number of the intron-gained genes (*p* = 3.3 × 10^−7^). Meanwhile, we found that the coding sequences length and gene length of the intron-gained genes are significantly longer than the conserved genes in *C. angaria* (*p* = 4.7 × 10^−22^ and 9.8 × 10^−15^, respectively).

One intron gain model is that the new intron was inserted into genomic DNA during DNA double-strand break repair [15]. Evidence supporting this model is the microhomology, or short, direct repeats flanking the gained introns, with one repeat positioned at the 5′ exon–intron boundary and the other repeat near the 3′ intron–exon boundary [15]. Therefore we compared the frequency of boundary-positioned microhomology between the conserved introns and the newly gained introns. Because the sample size is too small for species like *C. brenneri* and *C. briggsae*, we grouped all the 168 new introns as one sample in our comparison. The appearance of short, direct repeats was compared pairwisely between the conserved introns and the novel introns of the intron-gained genes. Short directed repeats ranging from three to eight base pairs were surveyed within ten bp sequences symmetrically across the exon–intron boundaries. The appearances of three to five bp short, direct repeats flanking the novel introns were significantly higher than those flanking conserved introns (Wilcoxon signed rank test, *p* = 1.4 × 10^−5^, 2.7 × 10^−4^, and 0.044, respectively). No significant differences were detected in the appearance of short, direct repeats ranging from six to eight bp (Wilcoxon signed rank test, *p* > 0.05 for all the three cases). No statistically significant differences in GC-content between the new introns and the conserved introns were detected (Wilcoxon signed rank test, *p* = 0.568). The novel introns do not have significantly different sizes from the conserved introns (Wilcoxon signed rank test, *p* = 0.548) but are substantially more proximate to the 3′ ends of genes (Wilcoxon signed rank test, *p* = 0.002).

### Gene ontology enrichment analysis of the intron-variant genes

Taking advantage of the wealth of genomic information in *C. elegans*, we characterized the intron-lost genes, intron-gained genes, and intron-conserved genes by gene ontology (GO) enrichment analysis, the cutoff *P*-value being set to 0.01 [66]. The significantly enriched GO terms are listed in Additional file 1: Table S7. Most of the significant terms are shared by the intron-lost and intron-conserved genes. Meanwhile, intron-lost genes are enriched in some particular GO terms, like ligase activity and ion binding. However, the 13 intron-gained genes do not enrich in any GO terms. Intron gains are unlikely related to specific functions.

## Discussion

With numerous studies on eukaryotic intron evolution, general patterns have been revealed. First, widespread heterogeneity in intron gain and loss rates has been repeatedly observed across both lineages and historical stages of the same lineage [5, 11, 14, 55, 67, 68]. Second, intron losses were generally more frequent than intron gains, with a few episodes of burst in the intron gain rates contributed by the amplification of transposable elements carrying splicing signals [6–20, 69]. Third, the intron losses of most lineages are precise removals of the intron sequences from chromosomal DNA. The cDNA-mediated intron loss model has been widely supported [44, 45, 50, 52, 54, 55, 70]. According to previous studies, the nematodes seem eccentric in their intron evolution. Their imprecise intron losses were reported to be outnumbered the precise intron losses, and most studies failed to observe the evidence supporting the cDNA-mediated model of intron loss, like preferential losses of adjacent introns and introns close to the 3′ end of genes, were not observed [50, 57–61].

Benefiting from the unprecedented availability of genomic sequences, we carried out a large-scale, comprehensive analysis of the intron evolution of nematodes. The risk of biased observations resulting from small samples could be minimized, and a general conclusion for the intron evolution of the phylum, Nematoda, has approached. By analyzing the 104 nematode genomes, we showed that, in the aspect of intron evolution, the nematodes are a typical rather than an eccentric group of eukaryotes. Their intron densities range from less than one to more than nine species, almost as wide as previously reported across all eukaryotes [1]. Significant heterogeneity in the rate of intron losses and gains has been observed across different nematode lineages and different evolutionary stages of the same lineage (Additional file 2: Fig. S2). Significantly more intron losses than intron gains were observed in the phylum-wide analysis and the in-depth analysis of the *Caenorhabditis* species. First, both intron-lost and intron-gained genes had longer coding sequences, longer gene sequences, and more introns. Then, We examined five aspects of lost introns that are generally believed as evidence supporting the model of cDNA-mediated intron loss. In the ten *Caenorhabditis* species, the dominance of the precise intron losses and high-level expression of the intron-lost genes were all fully confirmed. When the lost introns and the conserved introns were compared within each species, only some species exhibited significant differences in the preferential losses of short introns and the preferential losses of introns close to 3′-ends of genes. However, when the intron-lost genes from different species are considered together, the lost introns are significantly shorter and near the 3′-ends of genes than the extant introns of the same genes. As we see, the lacking of significance in some species should be attributed to the small sample sizes. Of course, the biased position and frequent loss of adjacent introns are not so strong as the other three aspects, precise intron losses, preferential loss of short introns, and high-level expression of the intron-lost genes. Although the 3′-biased position was initially suggested as evidence for the cDNA-mediated intron loss [48], it is not always observed with other evidence of the model [55, 71]. The present result highlights the importance of large sample size in intron evolution studies.

The mystery of intron gains left in *Caenorhabditis* is consistent with previous studies on other eukaryotic groups [13, 15, 54, 55, 72]. No possible source sequences have been identified for the 168 novel introns detected in *Caenorhabditis*. The source sequences are the molecular smoking gun in identifying novel introns [62, 73]. Although researchers failed to identify the source sequences of most novel introns, they could quickly identify orthologous introns by sequence similarity. As the novel introns should be gained after the divergence of the orthologous introns, there are several possible explanations for the failure in identifying source sequences [54]. The first is that the newly acquired introns diverge from their source sequences at an unexpectedly high rate. However, there is no evidence for the rapid divergence of recently gained introns in any eukaryotes. The second explanation is that the source sequences are in the dark matter that has not been sequenced. The discovery of introner elements as the novel intron sources is an example of finding a smoking gun from dark matter [17, 40, 42]. Meanwhile, an insight that could be learned from the studies of introner-elements contributed to novel introns is that the newly gained introns do not have an unexpectedly high divergent rate that makes them rapidly unrecognizable from their source sequences. It is also possible that the small number of intron gains, compared with a large number of intron losses, results from the imperfectness of the methods used to distinguish intron losses and gains. A minor technical error ratio might shift a pattern of exclusively intron losses to the observation of predominant intron losses with a few cases of intron gains. The last suspicion has been aggravated by the similarity between intron losses and gains in *Caenorhabditis*, like the observations of adjacent intron losses and adjacent intron gains, and the 3-biased positions of both lost introns and newly gained introns.

## Conclusions

Our large-scale analysis showed that the intron evolution dynamics of the nematodes and the mechanisms of intron loss and gains in *Caenorhabditis* are similar to that observed in most eukaryotic lineages. The abnormal pattern observed by previous studies should be attributed to the small samples analyzed. This study highlights the importance of a large sample in intron evolution studies and contributes to the coming consensus on the pattern and the mechanisms of intron losses and gains.

## Methods

We downloaded the genome sequences and annotation files of *D. melanogaster, H. sapiens*, and the 104 Nematoda species from Ensembl Metazoa 48, Ensembl 101, and WormBase (release WBPS14), respectively [63, 74, 75]. The accession numbers and genomic features of the species used in this study are shown in Additional file 1: Table S1.

### The orthologous genes

The orthologous genes of the 106 species were identified using BLAST v2.2.26 (using parameter blastall -p blastp -F F -e 1e-5 -m 8) [76]. The two-way best reciprocal BLAST hits were retained. Besides, only the orthologs present in over 90% (96/106) of species were used in the subsequent analyses. In total, 1577 sets of orthologs were obtained.

The ten *Caenorhabditis* species were selected for the in-depth analysis of intron evolution, including *C. angaria*, *C. brenneri*, *C. briggsae*, *C. elegans*, *C. japonica*, *C. latens*, *C. nigoni*, *C. remanei*, *C. sinica*, and *C. tropicalis*. The orthologs of *Caenorhabditis* were also identified using the two-way best reciprocal BLAST hits (E value threshold = 10^−10^) [76]. A total of 4892 sets of one-to-one orthologous genes were identified.

### The phylogenetic tree

We used CLUSTALW (version 2.1) [77] to align the protein sequences and the Gblocks program (version 0.91) to eliminate the poorly aligned regions [78]. One thousand five hundred fifty-one sets of orthologous genes were retained after the filtration. The identities of the multiple sequence alignments are presented in Additional file 2: Fig. S4. The filtered coding alignments were used to build the phylogenetic tree using RAxML (version 8.2.12) [79], with the parameters -f a -x 1533 -# 1000 -m GTRGAMMAX -s sequences.phy -q partitions.txt. The topology structure was displayed using iTOL [80].

### Inference of ancestral introns in nematode

We inferred the ancestral introns from 1577 sets of orthologs of the 104 nematode genomes using the MALIN package [65]. We generated a table of intron presence or absence in the orthologs using MALIN. It included 10,469 intron sites allowing a maximum of 11 ambiguous entries per site.

MALIN provides a variety of models to calculate the loss and gain rates and estimate intron content. The previous studies have shown that the model fit was significantly impacted by variations in loss rate across intron sites [5]. Moreover, inaccurate prediction of intron loss rate could lead to underestimating intron density of eukaryotic ancestors [12, 21]. In this study, intron loss and gain rates were optimized in MALIN using maximum likelihood with constant rate model and rate-variation model and starting from the standard null model, running 1000 optimization rounds (likelihood convergence threshold = 0.001). Each intron site has only a branch-specific gain and loss rate for the constant rate model. For the rate-variation model, intron loss = 2 and intron gain = 1. MALIN calculates gain and loss rates and intron content at the root by numerical optimization of the likelihood.

Then, we used MALIN to calculate the log-likelihood of the two models. We used 100 bootstrap rounds of the intron table to assess the uncertainty about the inferred rate parameters and the intron site histories for every node. For model comparison, the likelihood-ratio test statistic calculated as

$$\Delta =-2\times \left({L}_{1}-{L}_{2}\right)$$

The *L*_1_ is the log-likelihood of the constant rate model (*L*_1_ =  − 66,590), and the *L*_2_ is the log-likelihood of the rate-variation rate model (*L*_2_ =  − 65,107). The likelihood-ratio test statistic is 2967, which was then compared to *χ*^2^ distribution with *df* = 1, and the *p*-value is 0. Therefore, we rejected the constant rate model and chose the rate-variation model for calculating loss and gain rate.

Besides, we inferred the ancestral intron number of branch nodes by Dollo parsimony. Then, we scaled the number of inferred introns to intron density by,

$$Scaled\,intron\,density = N \times 3.65 \div 373$$

*N* is the present intron number by Dollo parsimony, and the 3.65 and 373 are intron density and intron number of *C. elegans* in the orthologous dataset, respectively. *C. elegans* was used as a reference because its genome has a high-quality annotation (Additional file 1: Table S2).

The rate variation model was also used to estimate intron site histories. Furthermore, we kept the intron site histories (intron loss or intron gain) with a posterior probability ≥ 0.99.

### Intron variation analysis in *Caenorhabditis*

The coding sequences of orthologs of *Caenorhabditis* species were aligned using CLUSTALW [77] and MUSCLE (version 3.8.31) [81]. The orthologous alignment's intron presence/absence state was compared using custom Perl scripts. Only when the introns present in all the ten species were designated a conserved intron position (6252 conserved intron positions). The candidate intron discordant positions must meet two constraints. Firstly, the gaps within 45 bp alignment sequences upstream and downstream of the intron variation positions were less than 10. Secondly, identities of the 45 bp alignment upstream and downstream the intron variation positions were more than 0.5. Besides, the intron-variation genes with at least one conserved intron position were retained.

Dollo and polymorphism parsimony algorithm (version 3.697) identified intron loss and potential intron gain, using parameter parsimony method = Dollo, and the input tree was shown in figure S2.

### Resampling analysis for the simultaneous loss of adjacent introns

We first calculated the probabilities of adjacent intron losses referring to Roy and Gilbert [50]*.* The probability that a gene losses adjacent intron is *Pr*{*d*|*l*,*r*}. *d*: pairs of lost adjacent introns (0 ~ *l*-1), *l*: lost introns, *r*: conserved intron and lost introns.

$$\Pr \{ d|l,r\} = \frac{{\left( {\begin{array}{*{20}c} {l - 1} \\ d \\ \end{array} } \right)\left( {\begin{array}{*{20}c} {r + 1} \\ {l - d} \\ \end{array} } \right)}}{{\left( {\begin{array}{*{20}c} {r + l} \\ l \\ \end{array} } \right)}}$$

We calculated the bias of adjacent intron loss of seven *Caenorhabditis* species (*C. tropicalis*, *C. brenneri*, *C. latens*, *C. remanei*, *C. sinica*, *C. nigoni*, and *C. briggsae*) (Table 4). For the other three species (*C. angaria*, *C. japonica*, and *C. elegans*), the number of lost-gene were too much to calculate.

Secondly, the probability of simultaneous loss of adjacent introns was estimated using the random sampling principle for ten *Caenorhabditis* (Table 4). For example, in *C. elegans*, there were 43 genes with > 1 lost-intron and 26 pairs of adjacent intron losses. The *C* represents the number of extant intron positions, and *L* represents the number of intron-lost positions. We randomly resampled *L* positions from all positions (*L* + *C*) and counted the pair number of adjacent positions for each gene. Then, the pair number obtained in resampling the 43 intron-lost genes was compared with the observed number 26. The null hypothesis is that intron loss was a random event, and so the pair numbers obtained in resamplings should be, on average, close to 26. On the contrary, if the pair number was much smaller than 26 in most rounds of resamplings, adjacent introns tend to be lost more frequently than randomly. The probability (*p*-value) was the ratio of the resampling times with equal or more adjacent positions (≥ 26 pairs) divided by the total resampling times (100,000). In 100,000 times random resampling, only 11 resampling results showed that adjacent pairs were higher than or equal to 26. As a result, the probability was 11 divided by 100,000 (1.1 × 10^−4^). It refused the null hypothesis, so the introns were not lost randomly at different positions.

### The representative lengths of introns

The length of a lost intron was represented by the length of its orthologous intron in the most closely related species, defined by the phylogenetic relationships shown in figure S2. For instance, the intron at the orthologous position of *C. remanei* was taken as a representative intron of the lost intron of *C. latens*. For the introns in *C. sinica*, the introns at orthologous positions in *C. briggsae* and *C. nigoni* were considered representative introns. The representative length of the *C. sinica* intron was the average length of the orthologous introns in *C. briggsae* and *C. nigoni*. In the length comparison between lost introns and conserved introns, representative lengths were also used for the conserved introns.

### Microhomology identification

Microhomology is defined as a pair of short, direct repeats around each end of an intron. Ten bp sequences symmetrically across the exon–intron boundaries of targeted introns between upstream and downstream were surveyed for the presence/absence of microhomology. We sequentially extracted the repeat sequences from the upstream and downstream boundary-positioned sequences and compared the similarities between the two regions. The repeat sizes were set from 3 to 8. Only the two sequences with entire consistency were regarded as homologous repeats.

### Analysis of RNA-seq data

With abundant food, optimal temperature (20 °C), and sparse population, the development of *Caenorhabditis* worms from embryo to adult can be divided into four larval stages, L1 to L4 [82]. We downloaded the RNA-seq data, SRR7781209 and SRR7781210 (L4-early adult stage), and SRR14578903, SRR14578904, and SRR14578905 (early embryos tissues) from the Sequence Read Archive data of the NCBI database [83]. The RNA-seq reads of *C. briggsae* (SRR7781208), *C. remanei* (SRR7781207, SRR7781212), and *C. brenneri* (SRR7781211), sampled from the L4-early adult stage were also downloaded from the same database. All these RNA-seq files are listed in Additional file 1: Table S8.

RNA-Seq reads were aligned to the reference genomes using TopHat algorithm v2.0.14 (using parameters—library-type fr-unstranded—min-segment-intron 10—max-segment-intron 20000) [84]. The mapped reads were used to re-annotate the exon–intron structures.

The RNA-seq count data of early embryos tissues (SRR14578903, SRR14578904, SRR14578905) were normalized to Fragments Per Kilobase per Million (FPKM) mapped reads using Cufflinks v2.2.1, an open-source software program, using the parameters—G-library-type fr-unstranded [85]. The FPKM values were used to represent gene expression levels.

### Statistical analysis

Data calculations were performed using a series of custom Perl scripts. Statistical analysis and plotting were performed using R v4.0.3 and SPSS R26.0.0. Chi-square test (chisq.test function), Mann–Whitney *U* test (wilcox.test), Benjamini–Hochberg test (p.adjust function), and PGLS were calculated using the R packages, phytools v0.7-70 [86], ape v5.4-1 [87], MASS v7.3-53 [88], mvtnorm v1.1-1 [89], and caper v1.0.1[90]. The phylogenetic signals were examined using phylosig functions (parameter method = lambda) in the R package phytools v0.7-70. Spearman rank correlation test and Wilcoxon signed ranks test were calculated using the SPSS. The plots were constructed using ggplot2 [91].

## Supplementary Information

**Additional file 1.** **Table S1.** The genomic and taxonomical information of the studied nematode species. **Table S2.** The predicted number and rate of intron losses and gains of each branch. **Table S3.** Correlations of intron length among Caenorhabditis species. **Table S4.** Data for Fig. 2. **Table S5.** Data for Fig. 3. **Table S6.** The detailed information of 168 cases of recently gained introns in Caenorhabditis. **Table S7.** GO annotations of the intron-lost, intron-gained, and intron-conserved genes in *C. elegans*. **Table S8.** The project IDs of the RNA-Seq data used in this study.

**Additional file 2.** **Figure S1.** Ancestral intron densities during the evolution of nematodes. **Figure S2.** Rates of intron losses and gains during the evolution of nematodes. **Figure S3.** Phylogenetic tree of Caenorhabditis andoutgroups. **Figure S4.** The identities of multiple sequence alignment.

## Data Availability

All data generated or analyzed during this study are included in this published article and its supplementary information files.

## Abbreviations

BH: Benjamini–Hochberg
BLASTP: Basic local alignment search tool for protein
CDS: Coding sequence (CDS)
FPKM: Fragments per kilobase of transcript per million mapped reads
GO: Gene ontology
NCBI: National Center for Biotechnology Information
PGLS: Phylogenetic generalized least squares
