# Supplementary material for: Intron losses and gains in the nematodes
Source: Biol Direct. 2022 Jun 5;17:13. doi: 10.1186/s13062-022-00328-8 (PMC9169325; doi:10.1186/s13062-022-00328-8)
Supplement: Supplementary file 2 — Additional file 2. Figure S1. Ancestral intron densities during the evolution of nematodes. Figure S2. Rates of intron losses and gains during the evolution of nematodes. Figure S3. Phylogenetic tree of Caenorhabditis andoutgroups. Figure S4. The identities of multiple sequence alignment. [file 13062_2022_328_MOESM2_ESM.docx]

**Intron losses and gains in** **the nematodes**

Ming-Yue Ma^1^, Ji Xia^1^, Kun-Xian Shu^*,1^, Deng-Ke Niu^*,2^

1 Chongqing Key Laboratory of Big Data for Bio Intelligence, School of Bioinformatics, Chongqing University of Posts and Telecommunications, Chongqing 400065, China

2 MOE Key Laboratory for Biodiversity Science and Ecological Engineering and Beijing Key Laboratory of Gene Resource and Molecular Development, College of Life Sciences, Beijing Normal University, Beijing 100875, China

*Corresponding authors:

Kun-Xian Shu, [shukx@cqupt.edu.cn](mailto:shukx@cqupt.edu.cn)

Deng-Ke Niu, dkniu@bnu.edu.cn.


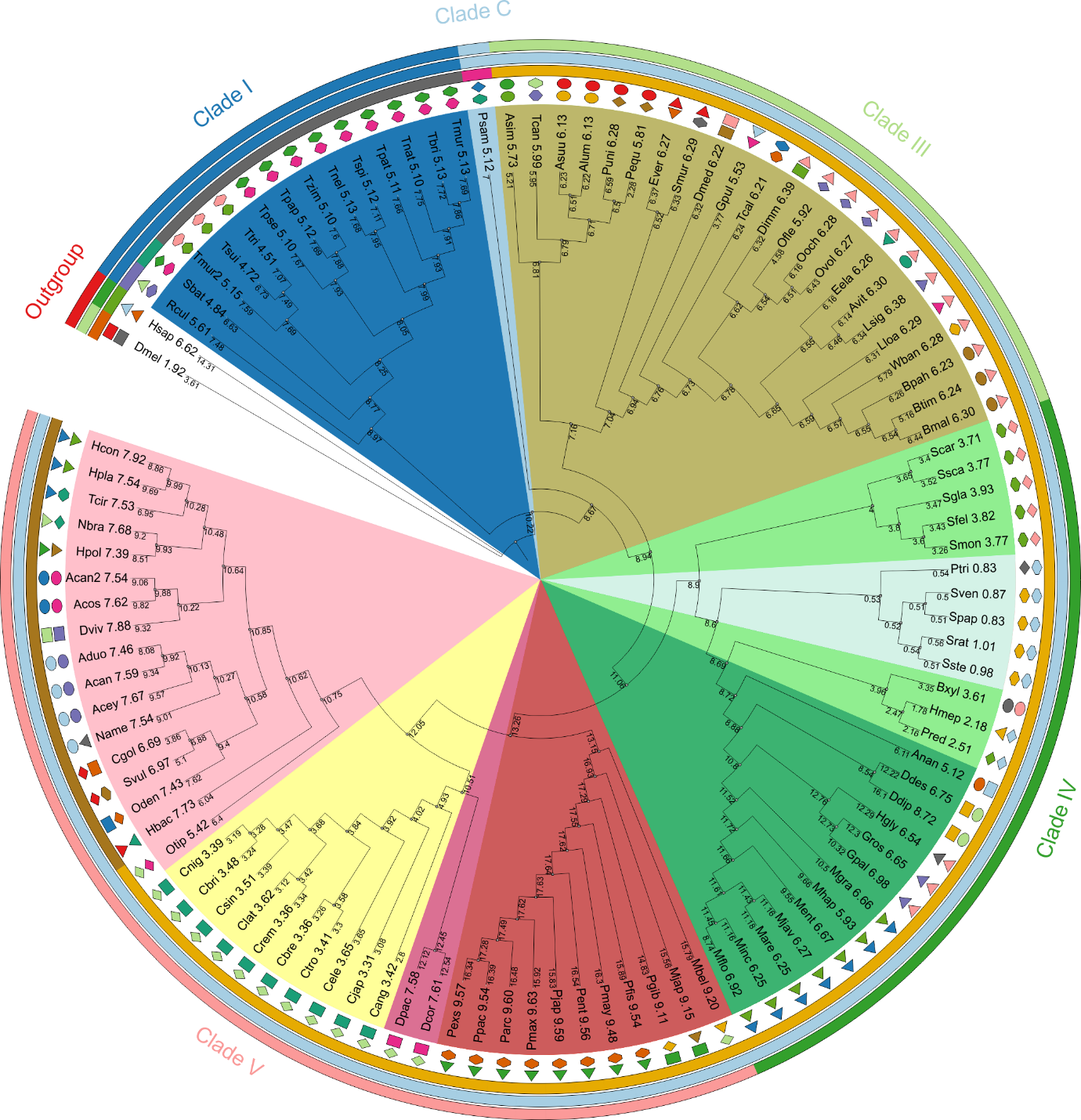


**Figure S1. Ancestral intron densities during the evolution of nematodes.**

The present tree is the best one obtained in the maximum likelihood analysis of 1,551 groups of orthologous genes. The ancestral intron number was inferred by Dollo parsimony and scaled the number of inferred introns to intron density by multiplying by 3.65 divided by 373. The 3.65 and 373 are intron density and number of reference species (C. elegans) in the orthologous dataset. Please see Table S2 for the full name of each species and the values present in this figure. Sister figures (Fig. 1 and fig. S2) show the number of intron losses and gains and the rates.


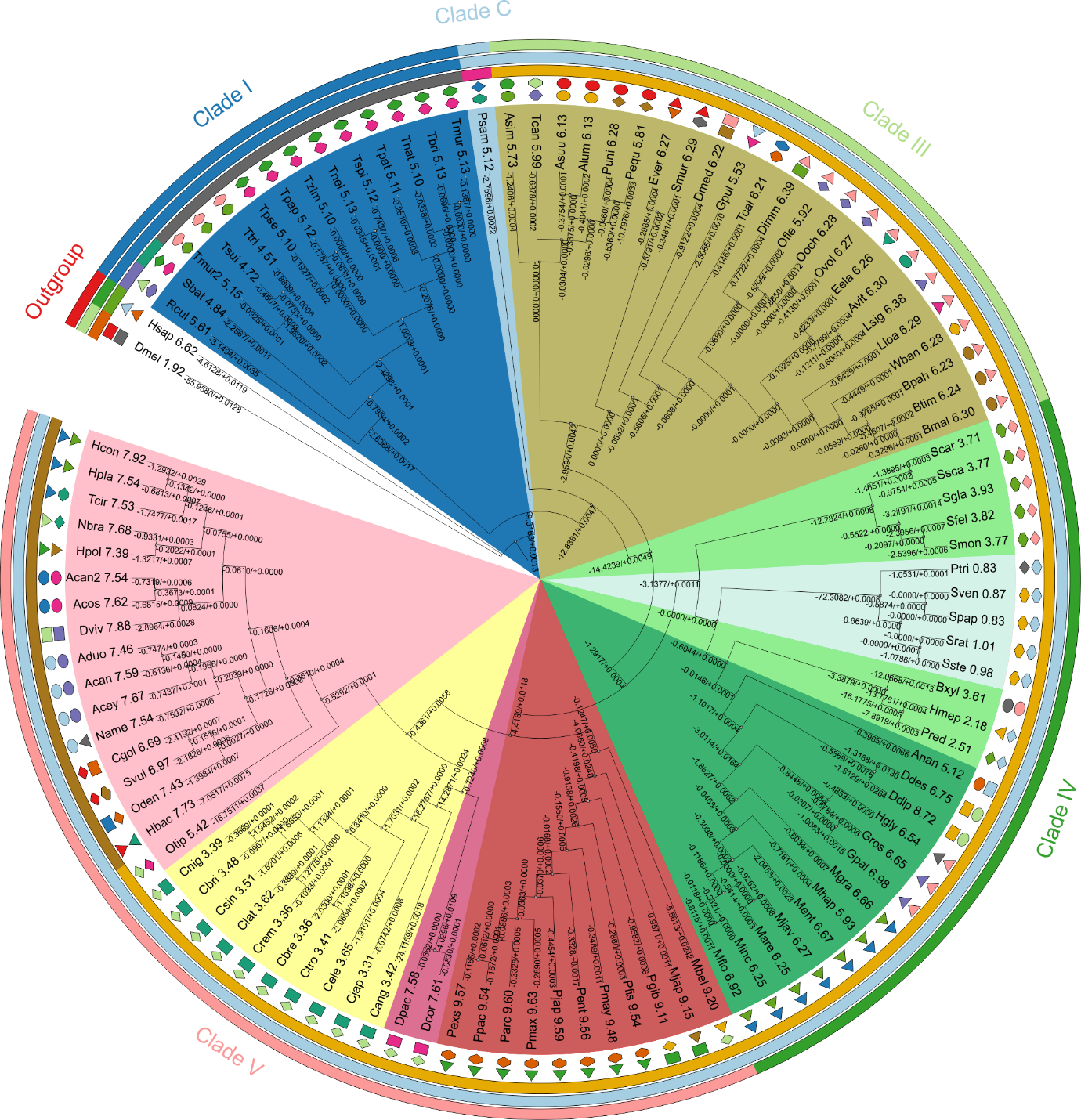


**Figure S2. Rates of intron losses and gains during the evolution of nematodes.**

The present tree is the best one obtained in the maximum likelihood analysis of 1,551 groups of orthologous genes. The rate was computed by maximum likelihood with the rate-variation model of MALIN. The values are displayed on the branch lines, using "+" and "-" symbols to represent intron gain and intron loss, respectively. Please see Table S2 for the full name of each species and the values present in this figure. Sister figures (Fig. 1 and S1) show the number of intron losses and gains and the ancestral intron densities.


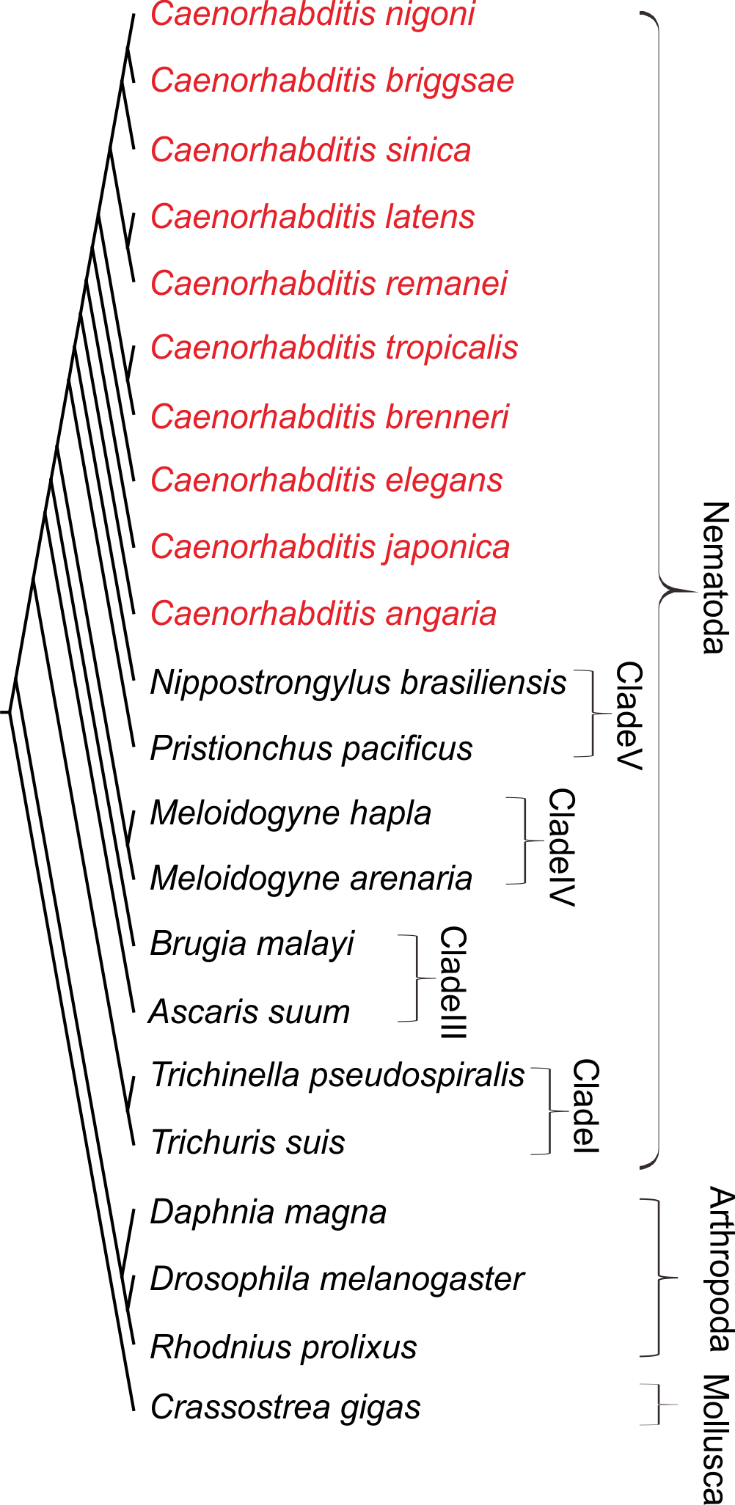


**Figure S3. Phylogenetic tree of *Caenorhabditis* and outgroups**

The phylogenetic tree among ten *Caenorhabditis* (shown as Red) and selected outgroup species, including the Clade I, Clade III, Clade IV and Clade V of nematode, and four other metazoa (*Rhodnius prolixus*, *Drosophila melanogaster*, *Daphnia magna*, *Crassostrea gigas*)


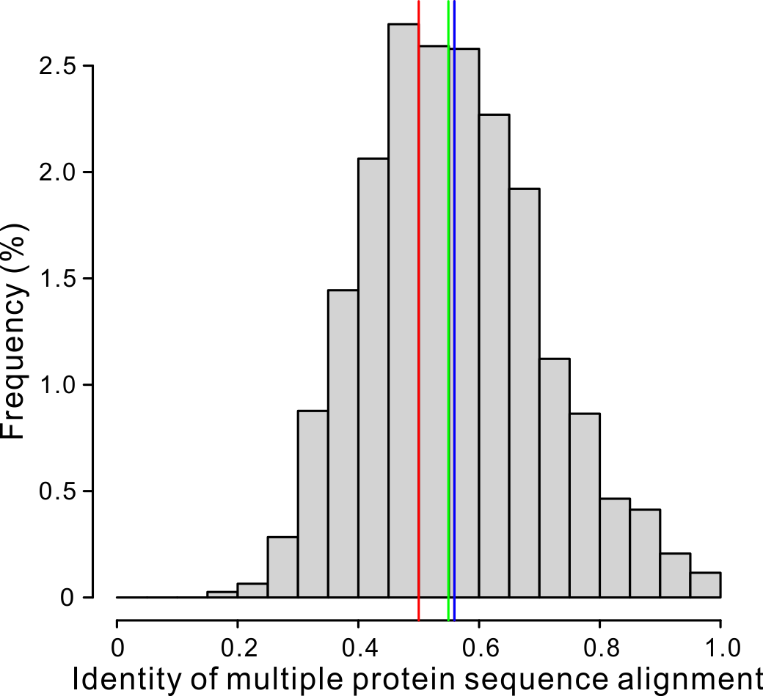


**Figure S4. The identities of multiple sequence alignment.**

The identities of 1551 groups of orthologous proteins in the nematodes. Redline is the *x* (identity) = 0.5. Greenline is the median value of the identities. Blueline is the mean value.
